# Hedgehog Is a Positive Regulator of FGF Signalling during Embryonic Tracheal Cell Migration

**DOI:** 10.1371/journal.pone.0092682

**Published:** 2014-03-20

**Authors:** Elisenda Butí, Duarte Mesquita, Sofia J. Araújo

**Affiliations:** 1 Developmental Biology Department, Institute of Molecular Biology of Barcelona (IBMB-CSIC), Barcelona, Spain; 2 Cell and Developmental Biology Programme, Institute for Research in Biomedicine (IRB Barcelona), Barcelona, Spain; Academia Sinica, Taiwan

## Abstract

Cell migration is a widespread and complex process that is crucial for morphogenesis and for the underlying invasion and metastasis of human cancers. During migration, cells are steered toward target sites by guidance molecules that induce cell direction and movement through complex intracellular mechanisms. The spatio-temporal regulation of the expression of these guidance molecules is of extreme importance for both normal morphogenesis and human disease. One way to achieve this precise regulation is by combinatorial inputs of different transcription factors. Here we used *Drosophila melanogaster* mutants with migration defects in the ganglionic branches of the tracheal system to further clarify guidance regulation during cell migration. By studying the cellular consequences of overactivated Hh signalling, using *ptc* mutants, we found that Hh positively regulates Bnl/FGF levels during embryonic stages. Our results show that Hh modulates cell migration non-autonomously in the tissues surrounding the action of its activity. We further demonstrate that the Hh signalling pathway regulates *bnl* expression via Stripe (Sr), a zinc-finger transcription factor with homology to the Early Growth Response (EGR) family of vertebrate transcription factors. We propose that Hh modulates embryonic cell migration by participating in the spatio-temporal regulation of *bnl* expression in a permissive mode. By doing so, we provide a molecular link between the activation of Hh signalling and increased chemotactic responses during cell migration.

## Introduction

During embryonic development, signalling pathways modulate cell behaviour by activating transcriptional programmes in response to extracellular signals. Over the past 50 years, it has been shown that surprisingly few pathways regulate these developmental programmes and that the dysregulation of these can lead to a plethora of human diseases, particularly to cancer. One characteristic of these developmental signalling systems is the selective transcriptional responsiveness of target genes to pathway activity. One major current challenge is to delineate the molecular mechanisms by which signalling pathways regulate cell movement and how this is dynamically coordinated during development.

Cell migration is a widespread and complex process that is crucial for morphogenesis and for the underlying invasion and metastasis of human cancers. Research into individual and collective cell migration, occurring under normal development or pathological conditions, is likely to yield clinically relevant insights. During collective cell migration, groups of cells migrate cohesively and are steered toward target sites by guidance molecules, stopping at the location where they are required for biological function. This requires activating target genes in their proper cellular context, while preventing their expression in other cells. Thus precise regulation of the expression of these guidance molecules is of extreme importance for morphogenesis and for human disease.

In *Drosophila melanogaster*, collective cell migration is present during tracheal development, an invertebrate model for tubulogenesis. Tracheal cell migration is a model of choice for studying how extracellular signals transduce into cellular movement [1]. During tracheal development, the main chemoattractant responsible for cell migration is the FGF homologue Branchless (Bnl) [2]. Bnl activates the FGF receptor (FGFR) Breathless (Btl) on tracheal tip-cells, which lead the concerted migration towards the Bnl source [3], [4]. *bnl* is expressed in a complex and dynamic pattern in tissues surrounding the developing tracheal system, thus controlling its migration and branching [2]. *bnl* expression is a determinant of the earliest branching events to the later programmes of tracheal gene expression. Two striking features characterise the expression of this gene during embryogenesis, namely its spatial complexity and its dynamic nature. However, very little is known about how the spatial and temporal control of this expression pattern is achieved. In addition, very few transcriptional regulators of *bnl* have been identified to date [5], [6], [7]. Therefore the major question remains as to how *bnl* cell-specific expression regulation is achieved.

The Hedgehog (Hh) signalling pathway is involved in embryonic morphogenesis, axonal guidance and angiogenesis [8]. Early studies of this pathway were based exclusively on genetic analysis of *Drosophila melanogaster*, though homologues were later found in many species where they were also found to play fundamental roles in development and disease [9]. Hh can act as both a short-range, contact-dependent factor, and a long-range, diffusible morphogen [8], [10]. The binding of Hh to its receptor Patched (Ptc) relieves Ptc-dependent repression of Smoothened (Smo) to activate downstream signalling events. Therefore, Ptc acts antagonistically to Hh, and *ptc*-loss of function phenotypes are similar to those observed when *hh* is overexpressed [8]. Downstream targets of Hh range from its own receptor Ptc and other signalling molecules like Decapentaplegic (Dpp) to transcription factors and cell cycle regulators [11]. Furthermore, activation of Hh signalling has been linked to several types of cancer [12].

The regulation of Hh signalling is crucial for the maintenance of proper gene expression in a variety of tissues during growth and cell survival, differentiation and migration [13], [14]. In addition, many targets of this pathway are expressed only in restricted domains within Hh-responsive tissues giving rise to the question of how tissue-specific responses are induced. This can be achieved by transcriptional control of target gene expression. According to current models, two ways of achieving this control are: i) by activator insufficiency, where a transcription factor alone is unable to strongly activate gene expression and/or ii) by cooperative activation, which combines signal-regulated transcription factors with local activators [15].

Here we used *D. melanogaster* mutants with migration defects in branches of the tracheal system to study guidance regulation during cell migration. By examining the functional consequences of overactivated Hh signalling, using *ptc* mutant embryos, we found that Hh modulates *bnl* transcription levels during embryonic stages. By doing so, this morphogen controls cell migration non-autonomously. We show that the Hh signalling pathway regulates *bnl* expression via Stripe (Sr), a transcription factor with homology to the Early Growth Response (EGR) family of vertebrate transcription factors. We propose that Sr participates in the regulation of *bnl* expression in a permissive mode, participating in the spatio-temporal control of its expression.

## Results

### *patched* mutant embryos have tracheal branch migration/extension defects

**Figure 1:**
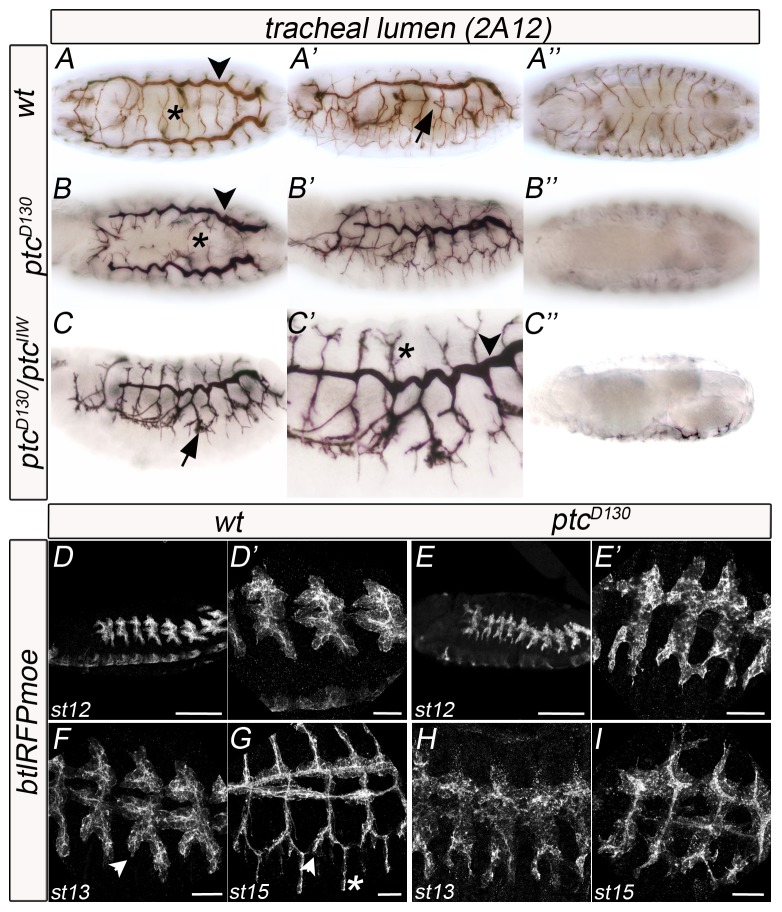
Ptc is required for tracheal morphogenesis. (A–C) Stage 16 wt (A) and *ptc* mutant (B–C) whole-mount embryos stained with the tracheal lumen antibody 2A12, using HRP immunohistochemistry for visualisation. A and B are dorsal views; A′, B′, C and C′ are lateral views and A″, B″ and C″ are ventral views. A, A′ and A″ are images of the same embryo; the same for B and C. Arrows indicate the visceral branch (VB), arrowheads the dorsal trunk (DT) and asterisks the dorsal branch (DB). (D–I) Lateral views of stage 12 to 15 wt (D, F, G) and *ptc* mutant (E, H, I) *btlRFPmoe* embryos stained with an anti-RFP antibody to visualise all tracheal cells. D′ and E′ are magnifications of D and E, respectively. Anterior is left in all panels; scale bars are 100 μm in D–E and 20 μm in F–I. Arrowheads indicate the lateral trunk anterior (LTa) and asterisks indicate the ganglionic branch (GB).

In *D. melanogaster* wild-type (wt) embryos, ganglionic branches (GBs) form at the lateral/ventral side and target the central nervous system (CNS) (Fig. 1A″). In an ethyl methanesulfonate (EMS) genetic screen for genes responsible for tracheal morphogenesis, we identified one mutant, D130, where GBs do not extend into the ventral nerve cord (VNC) and we focused our analysis on this phenotype (Fig. 1B″). However, defects in dorsal branch (DB) elongation (Fig. 1B and C′, asterisk) and in dorsal trunk (DT) thickness and convolution (Fig. 1B–C , arrowheads) were also observed. In addition, the visceral branches (VBs) were very often misplaced, localising more ventrally than in the wt (Fig. 1 A and C, arrows).

Using Bloomington Chromosome 2 deficiency kit, we found that allele D130 fails to complement Df(2R)ED1742, which deletes the region 44B8 to 44E3. Candidate gene analysis showed that D130 also failed to complement previously reported *patched* (*ptc*) mutant alleles. In addition, mutant embryos for the previously characterised *ptc* ^IIw^ and transheterozygote combinations of *ptc* ^D130^ with other alleles and Df(2R)ED1742 deficiency displayed the same GB phenotype (Fig. 1C″ and Fig. S1).

To confirm that *ptc* is affected in our D130 mutant, the *ptc* ^D130^ chromosome was sequenced, revealing a change of Q395STOP (Fig. S1). *ptc* encodes a 12-pass transmembrane domain protein, with two large extracellular domains, responsible for binding Hh, and a smaller intracellular domain, homologous to sterol-sensing-domain (SSD)-containing proteins. Amino acid (aa) 395 lies at the end of the first extracellular domain, just before the beginning of the SSD. *ptc^D130^* encodes a truncated protein (data not shown), which inferred by comparison to other alleles used in studies of Ptc function, is unable to bind and internalise Hh [16], [17], [18].

Tracheal development begins normally in *ptc* mutant embryos, as monitored by targeted expression of monomeric Red Fluorescent Protein (RFP) fused to moesin (mRFP::moesin, under the control of the *breathless* promoter, named btlmoeRFP reporter) in tracheal cells [19]. Placodes in *ptc^D130^* were correctly positioned and the first steps of cell migration occurred normally (Fig. 1E, E′). The initial steps of tracheal development were apparently regular until embryonic stage 12. At this point, we detected the first defects in GB extension. During stage 12, in wt and mutant embryos, the lateral trunk anterior (LTa) and the lateral trunk posterior/ganglionic branch (LTp/GB) migrated ventrally at the same level on the lateral side of the embryo (Fig. 1D′, E′). By stage 13, the wt LTp/GB (Fig. 1 F, asterisk) had advanced towards the ventral side of the embryo in relation to the LTa (Fig. 1 F, arrow). By wt stage 15, spatial separation between LTa and GB was evident (Fig. 1G, asterisk marks GB). Nevertheless, in *ptc* mutant embryos LTa and LTp/GB showed the same lateral elongation in the embryo until the end of embryogenesis, and we could not distinguish between LTp and GB, even at late stage 15 (Fig. 1 H, I).

Thus, *ptc* mutant GBs have migration/extension phenotypes indicating that *ptc* is required for proper GB morphogenesis in the tracheal system of *D. melanogaster*.

### *ptc* mutants have lower numbers of tracheal cells

**Figure 2:**
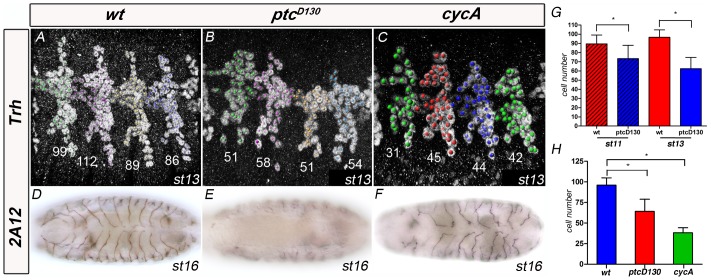
*ptc* mutants have fewer tracheal cells than wild-type. (A–C) Lateral views of Tr4-Tr7 of stage 13 wt (A), *ptc* mutant (B) and *cycA* mutant (C) embryos stained with anti-Trh antibody to visualise the tracheal nuclei. Nuclei were marked and counted using the Imaris software and the numbers in each metamere correspond to the number of nuclei marked. Anterior is left. (D–F) Ventral views of stage 16 wt (D), *ptc* mutant (E) and *cycA* mutant (F) whole-mount embryos stained with 2A12 antibody to visualise the tracheal lumen. GBs are present and enter the VNC in *cycA* mutant embryos, despite the very low numbers of tracheal cells. Anterior is left. (G,H) Quantification of tracheal cell numbers in wt, *ptc* and *cycA* embryos. (G) Comparison of cell numbers in Tr5 of *wt* and *ptc* mutant at stages 11 and 13; Tr5 at stage 11 have an average of 90 cells in the wt (n = 16) and 73 cells in *ptc* embryos (n = 18); Tr5 at stage 13 have an average of 97 cells in the wt (n = 11) and 63 cells in *ptc* embryos (n = 19). (H) Comparison of cell numbers in Tr5 of *wt*, *ptc* and *cycA* mutants at stage13; Tr5 at stage 11 have an average of 97 cells in the wt (n = 11), 63 cells in *ptc* embryos (n = 19) and 38 cells in *cycA* mutants (n = 8). *P-value≤0.01.

In the wt, no significant tracheal cell division or death is detected after embryonic stage 11, and tracheal branches maintain the same characteristic number of cells throughout embryonic development [20]. However, the Hh pathway has been shown to regulate cell proliferation and apoptosis during development [21], [22]. Thus, in *ptc* mutants, the lack of GB migration could be attributable to specific differences in cell proliferation or apoptosis in the tracheal system. Therefore, we examined the tracheal cell number in *ptc* mutant embryos and compared them with the wt (Fig. 2 A, B, G, H). From placode stages, *ptc* mutants showed a lower number of tracheal cells than wt embryos (Fig. 2 G). On average, 63 cells per tracheal metamere 5 (Tr5) of stage 13 embryos in *ptc* (n = 19) in comparison with 97 cells in Tr5 of wt embryos (n = 11) (Fig. 2 H). However, this lower cell number could not be correlated with a decrease in GB branch length, because in *cyclin A* (*cycA*) mutant embryos tracheal branches are formed and migrate to their destinations with only half the normal number of cells (38 cells, n = 8, Fig. 2C, H) and accomplish a standard tube diameter and length [23], with GBs invading the VNC as in the wt (Fig. 2 F). In addition, to rule out the possibility of specific cell death within the GB, we combined *ptc^D130^* with the H99 deficiency, which deletes the proapoptotic genes *reaper* (*rpr*), *head involution defective* (*hid*) and *grim*. In these mutant embryos, cell death no longer occurred, but they maintained the same phenotype as *ptc* mutants, with GBs not extending into the VNC (Fig. S1). Taken together, these results show that the *ptc* GB phenotypes are not attributable to differences in embryonic tracheal cell numbers.

### Specification of GB fate and the formation of tracheal cellular extensions is not dependent on Ptc activity

**Figure 3:**
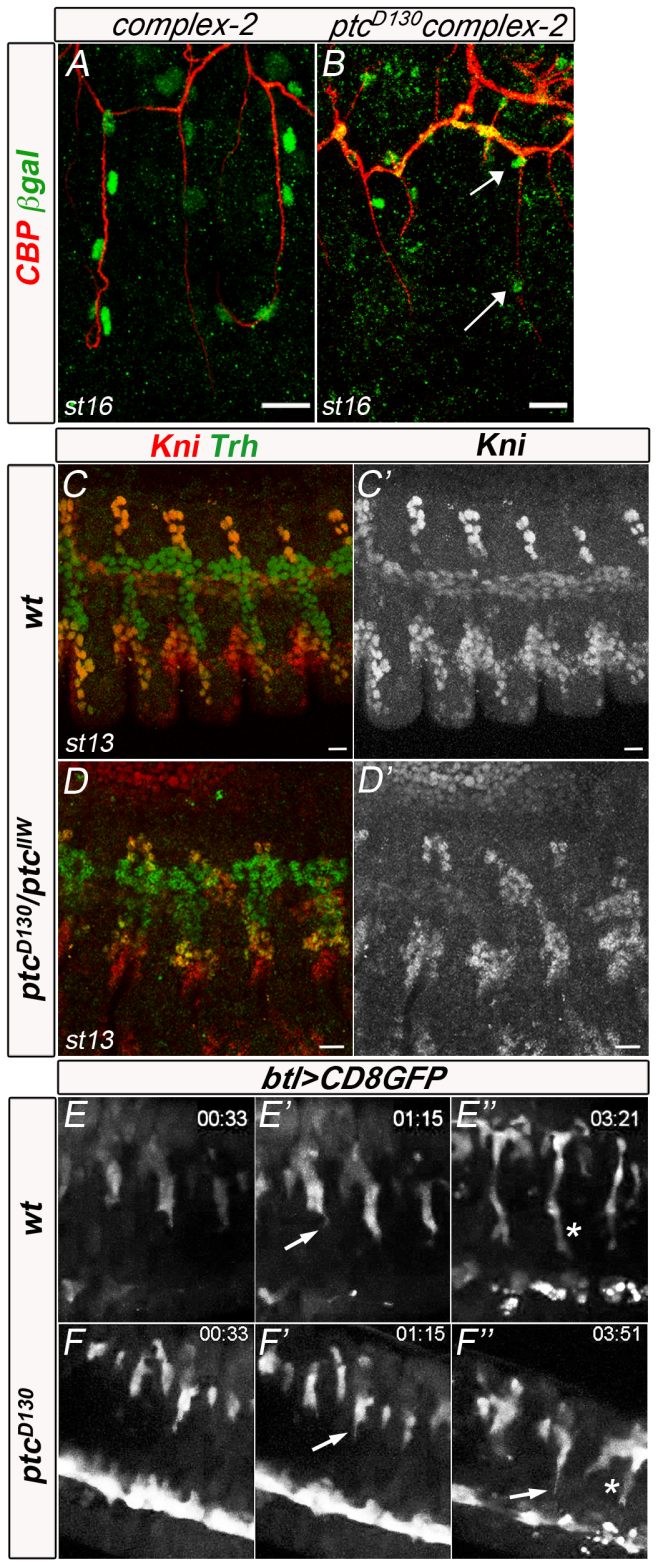
Ptc is not required for GB fate determination and the formation of tracheal cellular extensions. (A,B) Stage 16 wt (A) and *ptc* mutant (B) embryos carrying the Complex-2-lacZ reporter stained with the chitin binding probe (CBP) to visualise the tracheal lumen and the anti-ßgal antibody to mark all GB nuclei. Arrows indicate Complex-2 positive nuclei. (C–D) Stage 13 wt (C) and *ptc* mutant (D) embryos stained with anti-kni (red) and anti-trh antibody to mark all tracheal nuclei. C′ and D′ are images of the red-channel confocal projection. Kni expression is detected in cells of dorsal, visceral, lateral trunk and ganglionic branches, as well as in cells surrounding the LTa and LTp/GB in wt and mutant embryos. (E–F) Details of the lateral/ventral side of a live wt (E) and *ptc* mutant (F) embryo, marked with CD8::GFP driven by btlGAL4 to visualise cytoplasmic extensions during cell migration. Long cytoplasmic extensions are formed by the tip-cells in the wt and mutant GBs. All panels are lateral views. Anterior is left and scale bars are 10 μm in all panels.

By late embryogenesis, *ptc* mutants do not show any GBs extending into the VNC. How can Ptc affect this extension? One possibility could be that in *ptc* mutants tracheal cells of the LTp/GB cannot differentiate as proper GB cells and may adopt other cell fates. To check whether the absence of GBs in the VNC was caused by cell fate misspecification, we first analysed the presence of GB-specific markers in *ptc* mutant embryos. In the wt, GB cells express the specific marker Complex 2 [20] (Fig. 3 A). Therefore, we checked the presence of this marker in *ptc* mutant embryos. Despite the general disorganisation of the LTp/GB, Complex 2-expressing cells were detected in this branch (Fig. 3 B, arrows). This result indicates that GB cells express a proper GB cell marker. Therefore we can refer to the presence of these branches in *ptc* mutants, despite the lack of VNC invasion. Another explanation could be a change in branch fate induced by higher levels of Dpp in *ptc* mutants. Ptc induces *dpp* expression via Hh signalling (reviewed in [24]), and Dpp specifies tracheal ventral fates and dorsoventral migration via *knirps* (*kni*) activity [25], [26], [27]. To check if aberrant migration of GB cells in *ptc* mutant embryos is caused by changes in the Dpp signalling pathway, we monitored the expression of *kni* (Fig. 3 C, D). In *ptc* mutants, Kni protein localisation was unaffected in relation to the tracheal branches and was detected in the rudimentary dorsal, visceral, lateral and ganglionic branches (Fig. 3 D, D′). These observations rule out the possibility that aberrant tracheal migration in *ptc* mutant embryos is an indirect consequence of changes in GB cell fate.

Ptc may also affect GB cell migration by limiting the migratory capacity of these cells. The FGF homologue encoded by the *bnl* gene is expressed in cells adjacent to the tip of migrating branches and tracheal tip-cells react to this chemoattractant by forming dynamic filopodial extensions [28]. In order to check whether the absence of GB migration was due to a lack of filopodial extensions, we analysed the behaviour of *ptc* embryonic tracheal tip-cells *in vivo*. As the GB migrated towards the CNS, cell extensions were prominently seen in the wt GB tip-cell (Movie S1 and Fig. 3 E, arrow). In *ptc* mutants dynamic cell extensions were detected in the tip-cells (Movie S2 and Fig. 3 F, arrow). In addition, the cytoplasm of GB tip-cells elongated in both the wt and the mutant (Fig. 3 E–F, asterisks). These results demonstrate that, in the absence of functional Ptc, tracheal cells retain the capacity to form filopodial extensions.

### The *ptc* GB migration phenotype is associated with earlier tip-cell fate specification

The cell at the tip of each GB behaves as a leading cell and later differentiates as a terminal cell [7], [29]. The process of terminal cell differentiation requires the activity of the gene *blistered* (*bs*, also known as *pruned*) encoding the *Drosophila* Serum Response Factor (DSRF) [30], [31]. Three distinct DSRF enhancer elements were identified in *D. melanogaster*. One directs the expression in tracheal terminal cells and is regulated by *bnl*; and two were found to drive expression in wing imaginal discs and are regulated by *hh* [32].

**Figure 4:**
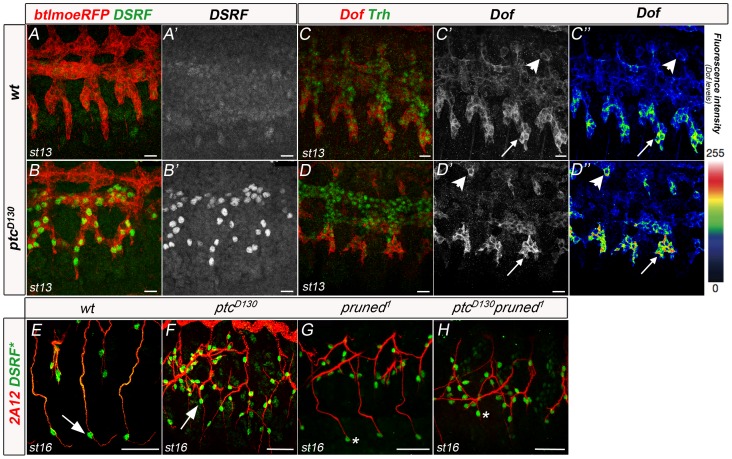
*ptc* mutants start expressing DSRF earlier. (A,B) Stage 13 wt (A, A′) and *ptc* mutant (B, B′) embryos carrying the btlmoeRFP reporter stained with anti-RFP to visualise the tracheal cells and anti-DSRF antibody to mark all terminal-cell nuclei. *ptc* embryos start expressing DSRF earlier than the wt. Scale bars are 10 μm. (C,D) Late stage 13 wt (C, C′) and *ptc* mutant (D, D′) embryos stained with anti-Dof and anti-trh antibody. All *ptc* embryos analysed have higher levels of Dof in DB tip-cells (arrowheads in D′ and D″, n = 12). Scale bars are 10 μm. (E,F) Detail of stage 16 GBs stained with 2A12 and anti-DSRF in wt (E) and *ptc* embryos (F). (G,H) Detail of stage 16 GBs from embryos carrying the pruned-lacZ reporter stained with 2A12 and anti-ßgal in *pruned* (DSRF) mutant (G) and double *ptc pruned* mutant. Terminal cells in *pruned* mutants do not extend their terminal lumen in wt or *ptc* mutant backgrounds (asterisks). Scale bars are 25 μm. DSRF* means that in panels E, F the presence of this protein is detected by the antibody, whereas in panels G,H terminal cells are marked by βgal expression. Anterior is left in all panels.

Thus, *ptc* mutant GB extension phenotypes could be attributable to defects in terminal cell fate specification. To further explore this hypothesis, we analysed DSRF expression in the GBs of *ptc* mutant embryos. LTa and LTp/GB cells started expressing DSRF earlier than in the wt (Fig. 4 A–B). In the latter, tracheal tip-cells began expressing DSRF, albeit at very low levels, at late stage 13 [33]. In *ptc* mutant embryos, DSRF expression was detected at early stage 13 and was fully established by late stage 13 (Fig. 4 B′ compare to same stage wt in A′). In wt and *ptc* mutant embryos, cells expressing DSRF behaved as terminal cells, extending a long intracellular lumen (Fig. 4 E, F, arrows).

DSRF induction in GB tip-cells is triggered by FGF signalling through activated MAPK [33]. The FGFR signalling cascade uses the adaptor protein Downstream-of-FGFR (Dof, also known as Stumps)[34], [35]. Dof expression is restricted to cells that express FGFRs where it is required to activate the MAPK cascade, specifically via FGF signalling [34]. Accordingly, we detected a stronger accumulation of Dof in cells of the LTa and LTp/GB of *ptc* mutant embryos in comparison with the same wt cells (Fig. 4 C–D). In the wt, Dof accumulated at higher levels only in the GB tip-cells (Fig. 4C′, arrow), whereas in *ptc* mutants, Dof was equally detected in both LTa and LTp/GB (Fig. 4D′–D″, arrow). In addition, accumulation in DB tip-cells at stage 13 was greater in *ptc* mutants (Fig. 4C′–C″and D′–D″ , arrowheads). This observation indicates that in *ptc* mutants, DB, LTa and LTp/GB cells have higher levels of FGFR pathway activation.

In order to study whether earlier levels of DSRF in tracheal tip-cells are responsible for the lack of migratory capacity of these cells towards the VNC, we generated double mutants for *ptc* and *bs* (Fig. 4 H). For this purpose, we used the *pruned^1^* null allele of *bs*, which has no functional DSRF, but expresses βgal in all *bs*-expressing cells [32] (Fig. 4 G). Analysis of these double mutants revealed that *ptc* mutant GB cells were still unable to migrate despite the absence of functional DSRF (Fig. 4 H). These results show that changes in DSRF expression in *ptc* mutants, or the earlier cell fate changes of these GB cells, are not responsible for their migratory impairment.

### Ptc is present in cells surrounding the GBs

**Figure 5:**
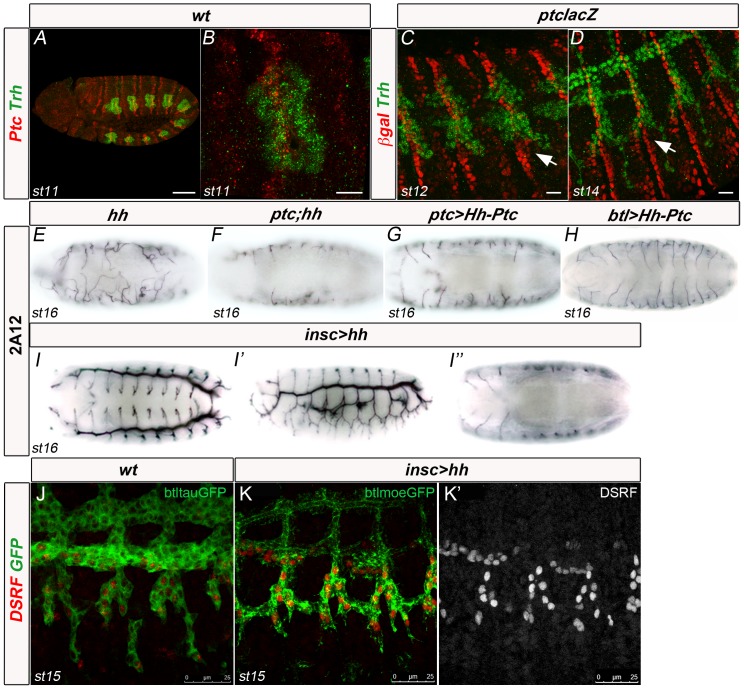
The Hh pathway modulates GB cell migration non-autonomously. (A,B) wt embryos stained with anti-Ptc and anti-Trh antibodies. Ptc protein is detected in vesicles in Ptc-expressing cells. (C,D) *ptclacZ* embryos stained with anti-ßgal and anti-Trh. Ptc expression is detected by nuclear ßgal presence. Scale bars are 50 μm in A and 10 μm in B–D. (E–H) Ventral views of stage 16 embryos stained with 2A12 to visualise the tracheal lumen. (I–I″) Dorsal, lateral and ventral views of the same stage 16 embryo expressing Hh in the VNC and stained with 2A12. Excess Hh in the VNC phenocopies the *ptc* mutant tracheal phenotype, but only on the ventral/lateral side of the embryo. (J–K) btl > tauGFP wt stage 15 embryo stained with anti-DSRF and GFP (J) and btlmoeGFP stage 15 embryos overexpressing Hh in the VNC and stained with anti-DSRF and GFP (K) or only DSRF (K′).

*ptc* is a *D. melanogaster* segment polarity gene detected throughout embryonic development, larval and pupal stages, with no reported maternal contribution [36], [37]. To define the domain of *ptc* expression in embryonic tissues during tracheal development, we used a *ptc-lacZ* enhancer trap line (Fig. 5 C, D) and a monoclonal antibody against the first extracellular domain of this receptor (Fig. 5 A, B), which in normal conditions detects Ptc predominantly in early endocytic vesicles [38]. According to our observations, *ptc-lacZ* ßgal expression mimics endogenous Ptc protein expression throughout all tracheal development stages (Fig. 5 A–D and Fig. S2). For this reason, we equally used these two approaches to detect Ptc. At stage 11, Ptc was detected in a stripe at the anterior portion of each tracheal placode (Fig. 5 A, B) [39]. Ptc was found in cells surrounding the migrating tracheal branches (Fig. 5 C and Fig. S2). Later on, Ptc was detected in cells surrounding the migrating GBs and LTps at stages 12 to 15 (Fig. 5 C, D, arrows and Fig. S2). In many cases, tracheal cells extended in very close contact to cells strongly expressing Ptc (Fig. 5 C, D). Using the Ptc antibody, we were unable to detect Ptc expression in GB cells from stage 13 (not shown). However, we could not discard the presence of Ptc in these cells at levels below detection by these methods.

### The Hh pathway modulates GB migration non-autonomously

To test whether the observed defects in GB migration are dependent on excess Hh signalling by lack of Ptc activity, we used an engineered form of Ptc, which is composed of the N-terminal signalling portion of Hh joined via HA tags to the Ptc receptor [40]. Named Hh-Ptc, this construct behaves as a cell-autonomous form of Ptc constitutively-bound to Hh, activating the Hh pathway in all cells where it is expressed [40]. When overexpressed in the *ptc* domain of expression, using a *ptcGAL4* driver, Hh-Ptc induced defects similar to those detected in the absence of Ptc (Fig. 5 G). In addition, expression of a Ci-activated form in Ptc-positive cells also generated a defective GB phenotype (Fig. S2). These experiments confirmed that activation of the Hh pathway in Ptc-positive cells is responsible for the migration phenotypes observed. In order to test whether the effects of Ptc on the GB phenotypes derived from within the tracheal cells, we expressed the same Hh-Ptc in all tracheal cells using *btl*GAL4 (Fig. 5 H). This ectopic expression did not induce a significant tracheal phenotype, thereby confirming a non-autonomous effect of Hh signalling in GB patterning that is not mediated by the activation of this pathway in tracheal cells. In order to check whether inhibition of GB migration was due to excess Hh signalling in cells surrounding this branch, we misexpressed *hh* in the VNC of wt embryos, using an *inscGAL4* driver [7]. Consistently, this misexpression resulted in the abrogation of GB migration/extension and in earlier expression of DSRF, like in *ptc* mutant embryos, because of increased Hh signalling in cells within and surrounding the VNC (Fig. 5 K).

In addition to binding Hh to initiate signalling, Ptc also modulates the extracellular gradient of Hh [38], [41]. To analyse whether changes in this gradient in the cells surrounding tracheal cells contributed for the GB phenotype, we analysed GB migration in double mutants for *ptc* and *hh* (Fig. 5 F). In *ptc;hh* embryos GBs behaved like *ptc* embryos. This observation sustains that changes in the Hh gradient are not responsible for the observed phenotype and confirms that the phenotypes are due to increased levels of Hh signalling. In *hh* mutants, when Hh signalling was inhibited, GBs still invaded the VNC despite the overall morphogenetic defects (Fig. 5 E). In addition, when the Hh pathway was silenced in Btl- or Ptc-positive cells by expressing a Hh-insensitive form of Ptc, PtcΔloop2, which constitutively inhibits Smo [18], GB extension was not affected (Fig. S2).

Taken together, these data indicate a non-autonomous effect of *ptc* in GB migration/extension, mediated by increased Hh signalling in cells surrounding these tracheal branches.

### Hh signalling regulates *bnl* transcription

Like all tracheal branches, GB migration depends on the dynamic expression of the FGF homologue *bnl*. Both the absence and excess of *bnl* expression can inhibit branch extension/migration [2]. In *ptc* mutant embryos, we observed earlier DSRF expression and higher levels of Dof in all LTa and LTp/GB (Fig. 4D′ ). For these reasons, we questioned whether the GB phenotypes observed in *ptc* mutants could be attributed to changes in *bnl* expression.

**Figure 6:**
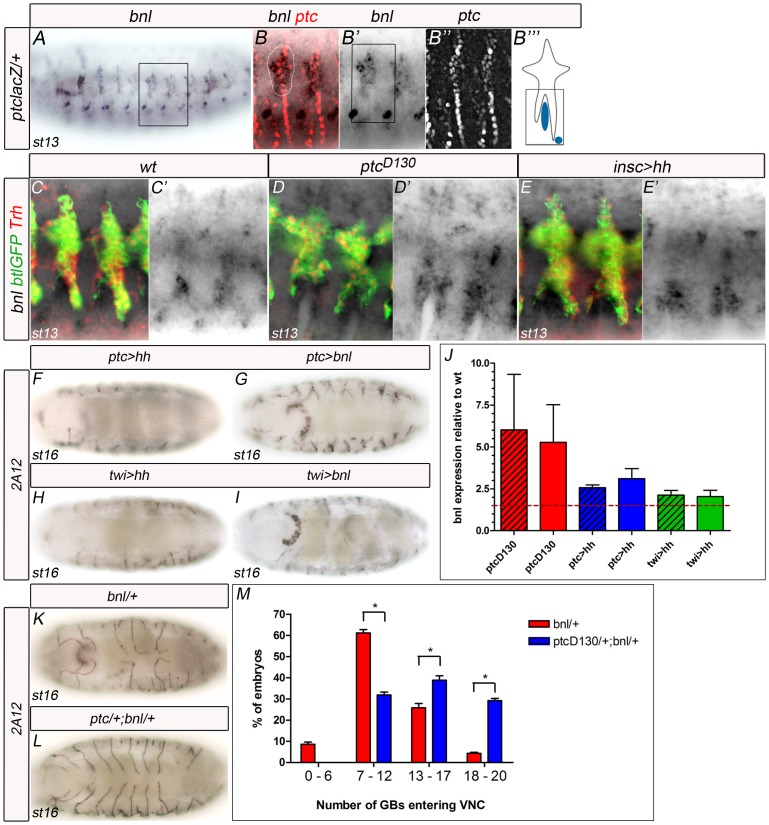
The Hh pathway modulates Bnl expression. (A,B) *bnl* expression in a *ptclacZ* heterozygous stage 13 embryo also stained with lacZ. (B) Marked area contains *ptc*-expressing cells that co-express *bnl* in cells surrounding the migrating GBs. (B′) Only *in situ bnl* expression; Rectangle marks the are depicted in panel B′′′ for clarity. (B″) Only ptclacZ expression. (B′′′) Schematic representation of the main areas of *bnl* expression surrounding LTa and LTp/GB. (C–E) *bnl* expression in wt (C), *ptc* (D) and in embryos expressing Hh in the VNC (E), in stage 13 embryos co-stained with anti-GFP and anti-Trh to mark all tracheal cells. C′, D′ and F′ show only the *bnl in situ* hybridisation pattern. (F–I) Ventral views of stage 16 embryos stained with 2A12 to visualise the tracheal lumen. (J) Graphic representation of quantitative Real-Time PCR results. The two columns with the same colour represent independent experiments using the same genotype. Hashed red line marks 1.5-fold expression. All overexpression experiments induce greater levels of *bnl* expression than those in wt. P-values ≤0.01. (K,L) Ventral views of stage 16 embryos stained with 2A12 to visualise the tracheal lumen. (M) Quantification of the number of GBs entering the VNC in embryos heterozygous for *bnl* (red) in comparison with embryos transheterozygous for *ptc* and *bnl* (blue) *P-values ≤0.01.

For Hh signalling to be able to induce *bnl* mRNA, Ptc is required in the cells that normally express *bnl*. Indeed, we detected Ptc in *bnl*-expressing cells surrounding the GB (Fig. 6 A–B, arrows), thus confirming that these cells could activate Hh signalling.

In *ptc* mutants, *bnl* mRNA levels were higher in cells surrounding the tracheal LTa and LTp at stage 13 and the same observation was made when *hh* was overexpressed in the VNC (Fig. 6 D–E). In addition, when *UASHh-Ptc* was expressed in *ptc*-expressing cells (Fig. 5 G), the effect on GBs was similar as to when *UASbnl* was overexpressed using the same driver (Fig. 6 G). Moreover, overexpression of Hh or Bnl using *ptcGAL4* or *twiGAL4* phenocopies the *ptc* mutant GB migration/extension phenotype (Fig. 6 F–I).

To more clearly assess the role of the Hh pathway as a regulator of *bnl* transcription, we analysed the levels of *bnl* transcripts by quantitative Real-Time PCR (qRT-PCR) in whole embryos from stage 12. Homozygous *ptc* embryos showed an average of 5.5-fold increase in the expression of this gene. Consistently, we also observed a 3-fold increase in *bnl* transcription upon overexpression of *hh* using *ptcGAL4*. Upon ectopic expression of *hh* using *twiGAL4*, a driver expressing in the mesoderm from stage 6 [42], a 2-fold increase in *bnl* expression was detected (Fig. 6 J). As a positive control, we found a 14.5-fold increase in *bnl* mRNA levels upon ectopic expression of *bnl* using the *inscGAL4* line (Table S1 in Tables S1). Taken together, these results confirm that higher levels of Hh in cells near the GB and/or activation of the Hh pathway in cells surrounding the GB increase *bnl* transcription levels.

Taken together, these results demonstrate that lack of functional Ptc or overactivation of the Hh pathway induces higher levels of *bnl* expression in *ptc-*expressing cells.

### The Hh signalling pathway is epistatic to *bnl*

In order to further ascertain the upregulation of *bnl* expression by the Hh pathway, we analysed the genetic interaction between *ptc* and *bnl*. *bnl* is a haploinsufficient locus whose products are present at limiting concentrations in heterozygous (*bnl*/+) individuals. More than 60% of heterozygous *bnl* embryos show occasional missing or stalled branches with the GBs most often affected [2]. Using *ptc* heterozygous embryos, we analysed whether these phenotypes are altered by modifying the Hh pathway. We quantified how many GBs entered the VNC in embryos trans-heterozygous for *ptc* and *bnl* (n = 48) and detected a consistent increase in comparison with *bnl* heterozygous embryos (n = 115) (Fig. 6K–M ). The total average of GBs entering the VNC in *bnl* heterozygotes increased from 11 to 15 by removing only one copy of *ptc*. This increase was even clearer when the numbers of GBs entering the VNC were divided into four groups, as shown in Fig. 6 M. Most of the embryos (68.1%) transheterozygous for *bnl* and *ptc* had from 13–20 GBs entering the VNC, in contrast to *bnl* heterozygous embryos, which were only 30.2% in this group. Therefore we can conclude that *ptc* genetically interacts with *bnl* and that higher levels of Hh signalling can overcome the GB migration defects present in *bnl* heterozygous embryos.

### Hh signalling regulates *bnl* transcription through the induction of *stripe* (*sr*)

We then addressed whether this regulation of *bnl* expression by the Hh signalling pathway was direct or through an intermediate regulator of *bnl* expression. Of the many studies on the downstream effectors of the Hh pathway, none has found Bnl/FGF to be a direct target of the pathway. In addition, little is known about the upstream mechanisms that establish the dynamic expression pattern of *bnl*.

**Figure 7:**
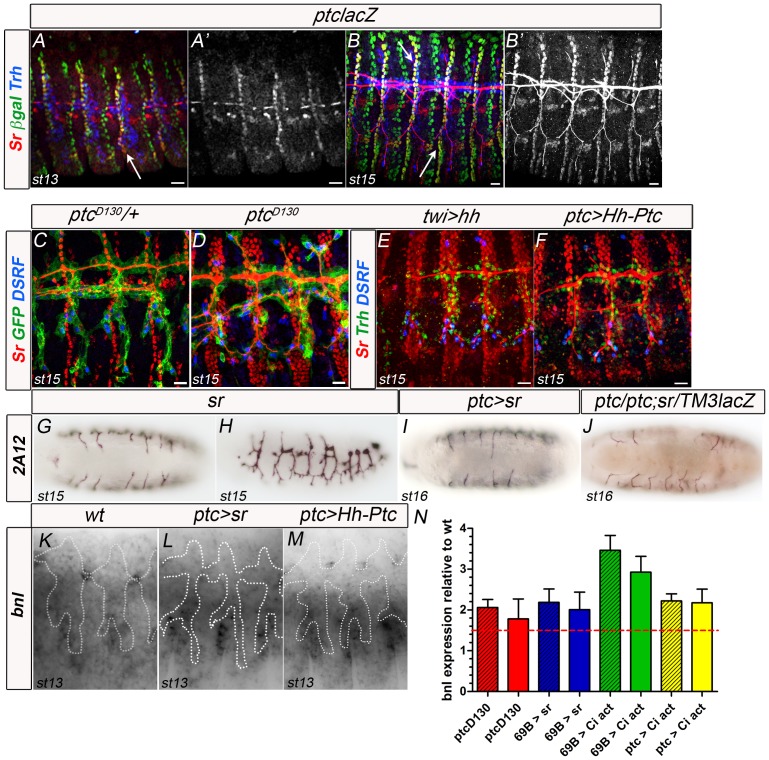
Hh signalling regulates *bnl* transcription through the induction of *stripe* (*sr*). (A, B) Stage 13 (A, A′) and *15* (B, B′) embryos carrying the ptclacZ reporter stained with anti-βgal, anti-SrB antibody and anti-Trh. Sr is expressed in some *ptc*-expressing cells, in particular cells surrounding the Ltp/GB (arrows). At late stages, anti-SrB antibody is unspecifically detected in the tracheal lumen. Scale bars are 10 μm. (C, D) Stage 15 *ptc* heterozygous (C) or homozygous (D) embryos stained with anti-SrB, anti-GFP and anti-DSRF. The Sr expression domain is enlarged in *ptc* mutant embryos. Scale bars are 10 μm. (E, F) Stage 15 embryos overexpressing *hh* in *twi*-expressing cells (E) and overexpressing Hh-Ptc in *ptc* expressing cells (F) stained with anti-SrB, anti-Trh and anti-DSRF. The Sr expression domain is enlarged in both overexpression experiments. Scale bars are 10 μm. (G, H) Ventral and lateral view of a stage 15 *sr* mutant embryo stained with 2A12 to visualise the tracheal lumen. (I) Ventral view of a stage 16 embryo overexpressing *sr* in *ptc*-expressing cells stained with 2A12 to visualise the tracheal lumen. (J) Ventral view of a stage 16 *ptc* mutant embryo heterozygous for *sr* stained with 2A12 to visualise the tracheal lumen in black and βgal to mark the TM3ftzlacZ balancer in brown; midline cells are marked in brown. (K, L, M) *bnl* expression in stage 13 wt embryos (G), embryos expressing Hh-Ptc (H) and expressing *sr* (I) in *ptc*-expressing cells. Marked area contains *trh*-expressing cells (from another channel, not shown). Anterior is left in all panels. (N) Graphic representation of quantitative Real-Time PCR results. The two columns with the same colour represent independent experiments using the same genotype. Hashed red line marks 1.5-fold expression. All overexpression experiments induce greater levels of *bnl* expression than those in wt. P-values ≤0.01.

*stripe* (*sr*) encodes a zinc-finger transcription factor of the early growth response (EGR) family implicated in tendon-cell differentiation [43]. Its dynamic expression is determined by the interplay between Hh and Wg [44] and is required for the proper migration of myotubes and tracheal branches [43], [45], [46]. In addition, *sr* is expressed by *bnl*-expressing cells, and *sr* null mutants display tracheal migration phenotypes (Fig. 7 G, H and [46], [47]). Therefore, we examined whether *sr* could be the effector of Hh signalling responsible for modulating *bnl* levels during tracheal migration. In order to be such an effector, *sr* needs to be present in *ptc*-positive cells and in particular in the same cells that express *bnl*. Therefore we analysed the co-expression of *sr* and *ptc* in wt tracheal development stages and concluded that indeed *sr* is present in cells expressing *ptc* (Fig. 7A–B , arrows). Then, we analysed *sr* expression in *ptc* mutant embryos and in embryos overexpressing *hh* under the control of *twi*, in which we had previously detected a phenotype similar to that of *ptc* and also higher levels of *bnl* expression (Fig. 6). In both cases, broader domains of *sr* expression in association with defective GB migration phenotypes were observed (Fig. 7 C, D and E). In addition, cell autonomous overactivation of the Hh pathway by means of Hh-Ptc expression led to higher levels of *sr* induction (Fig. 7F ) associated with greater *bnl* expression in cells surrounding the LTa and LTp/GB (Fig. 7 G) and defective GB migration (Fig. 5 G). To conclude, we analysed GB migration and *bnl* expression in embryos overexpressing *sr* in the *ptc* domain, which also causes a broader ectodermal *sr* expression domain [45]. These embryos showed strong tracheal migration phenotypes from early stages, ranging from lack of dorsal trunk fusions to general DB and GB migration phenotypes (Fig. 7 I). Furthermore, higher levels of *bnl* expression in cells surrounding the migrating GBs were detected (Fig. 7 L). These results show that higher levels of Hh signalling induce broader domains of *sr* expression and that these are correlated with greater *bnl* expression in cells surrounding the GB.

If this is the case, reduction of *sr* dosage in a *ptc* genetic background should improve the migratory capacity of GB cells whereas complete removal of Sr should have the same phenotype either alone or in a *ptc* genetic background. Indeed, analysis of double mutants for *ptc* and *sr*, proved this to be correct. Removing one copy of *sr* in a *ptc* background rescues the migration of many GBs (Fig. 7 L), whereas embryos mutant for the two genes have impaired GB migration, like *sr* and *bnl* mutants (Fig. 7 I, J and not shown). We confirmed the effect of Sr on *bnl* expression by analysing the levels of *bnl* transcripts by quantitative Real-Time PCR (qRT-PCR) in whole embryos from stage 12. We observed a 2-fold increase in *bnl* transcription upon overexpression of *sr* in the epidermis using *69BGAL4* (Fig. 7 N and Table S2 in Tables S1). Taken together, these results show that increasing sr expression in embryos increases *bnl* transcription levels.

Finally, to confirm that upregulation of *bnl* expression is dependent on Ci, we analysed *bnl* levels in Ci overexpressing embryos. We observed a 2-fold increase in *bnl* transcription in embryos overexpressing Ci in the *ptc* domain and a 3-fold increase in embryos overexpressing Ci in the epidermis using *69BGAL4* (Fig. 7 N and Table S2 in Tables S1).

Altogether, these results indicate that the Hh pathway modulates GB migration by regulating Bnl via Sr, in a Ci-dependent manner.

## Discussion

Hh and FGF signalling play a key role in normal embryonic development. In many tumour settings these pathways are dysregulated, and they are also crucial for driving tumour formation and angiogenesis [12], [48]. Due to the relevance of these signalling cascades during cell migration in various biological contexts, it is crucial to unveil the upstream and downstream events that lead to their spatio-temporal regulation. Here we show that the activation of Hh signalling increases *bnl* expression levels in *D. melanogaster* embryos. Other model systems also show a dependence of various FGFs on Hh signalling [49], [50], [51], [52], [53], [54]. However, these connections have only been reported to affect proliferation/growth and not cell movement.

**Figure 8:**
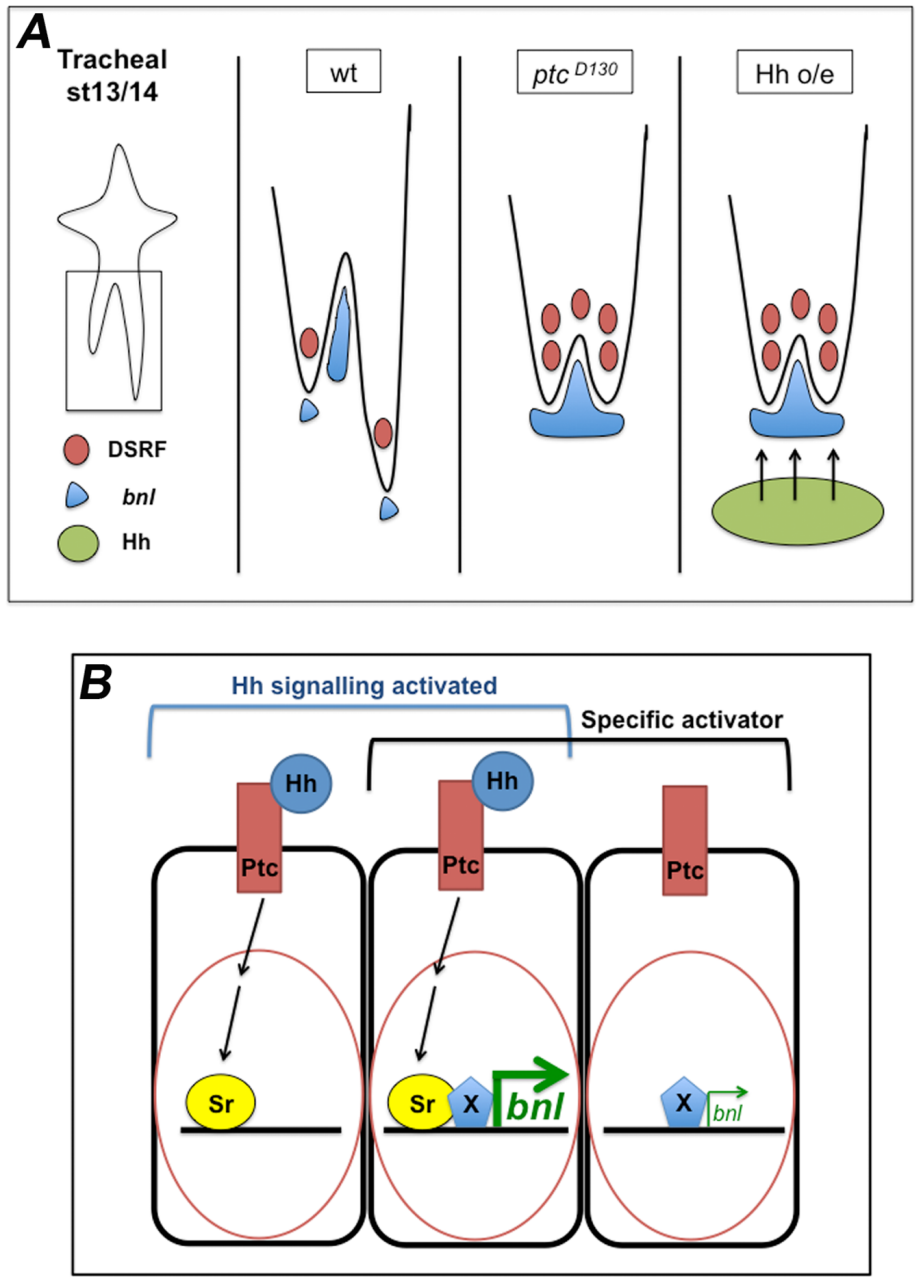
Proposed model of the role of Hh signalling in the regulation of *bnl* expression. (A) Model of changes in *bnl* expression dependent on Hh signalling in cells surrounding the tracheal tip-cells. Drawn are the LTa and LTp/GB in the wt, *ptc* mutant and Hh overexpression scenarios. Both the absence of Ptc activity and overexpression of Hh in cells near the migrating LTp/GB have the same inhibitory effect on GB migration. This is due to higher levels of *bnl* expression induced by the Hh pathway. (B) Proposed model for the activation of *bnl* expression via Sr and a cell/tissue-specific co-activator. When Hh signalling is active, SrB is one of its target genes being expressed in *ptc*-expressing cells. When these cells also express a specific *bnl* activator (X), *bnl* expression is induced at high levels. In the absence of Sr, X can drive *bnl* expression but only at low levels. The activation of the Hh pathway in addition to the expression of specific local activators is responsible for the fast upregulation of *bnl* expression in the tissues surrounding the migrating tracheal cells. According to this model, Sr is involved in the positive regulation of *bnl* expression in a permissive mode.

Hh is present throughout *D. melanogaster* embryonic development where it affects numerous cellular processes [8]. Bnl/FGF signalling is used repeatedly during tracheal development, where branch outgrowth is directed toward *bnl*-expressing non-tracheal target tissues via chemotactic induction of cell migration [2]. Here we analysed tracheal cell migration in conditions where the Hh pathway is overactivated. This allowed us to detect downstream events that are not evident when using Hh mutant phenotypes. Loss of function situations are not as effective in identifying redundant genes or different partners in cooperative transcription regulation of target gene expression. By analysing embryos in Hh gain of function conditions we found that Hh signalling is associated with the modulation of Bnl/FGF levels. In addition, we identified Sr as an upstream regulator of Bnl/FGF expression in cells surrounding the GB and showed the importance of Hh signalling for its expression (Fig. 8).

### Hh and tracheal cell migration

Previous lines of evidence have indicated that Hh plays an active role in tracheal development. In *hh* mutants, six to eight cells remain on the ectoderm after invagination, and tracheal placodes have fewer cells [39]. We also found that in *ptc* mutant embryos, fewer cells account for the tracheal tree at later stages, independently of apoptosis. These observations suggest that Hh signalling affects tracheal cell fate or proliferation, although *trachealess* (*trh*) expression is not dependent on *hh* [11].

From embryonic stage 12 onwards, the most severe tracheal defect previously observed in *hh* mutant embryos is an impairment in cell migration. And at later developmental stages, the Hh pathway was shown to be required for patterning terminal cells in the dorsal branches [39]. These authors also reported that compromising the activity of the pathway reduces the extent of terminal branching, while activation of the Hh pathway gives rise to an excess of terminal cells in the dorsal branch [39]. Hh has also been shown to act on terminal cells during their cytoplasmic extension, exerting its attractive effect from the epidermis [55]. In the light of our results, we attribute these Hh loss- and gain-of-function phenotypes to the lower or higher levels of *bnl* expression present in *hh* mutants or when the pathway is overactivated, respectively.

Here we demonstrate that Hh modulates tracheal cell migration non-autonomously by regulating *bnl* expression autonomously. Overactivated Hh signalling leads to higher levels of *bnl* in *ptc*-expressing cells. Higher levels of this chemoattractant in tissues surrounding the tracheal tip-cells have the same effect as inhibition of *bnl* expression [2]. This is consistent with the classical view that chemotaxis follows a bell-shaped curve in which excess ligand inhibits cell movement [56]. More precisely, here we have shown that overexpression of *bnl* has the same effect as the absence of *bnl* expression in limiting GB migration towards the VNC.

This upregulation of *bnl* expression also leads to an earlier expression of DSRF in tracheal cells. Besides, since *ptc* mutant trachea have less cells this also means that proportionately more cells express DSRF. In addition, it can be expected that higher levels of Bnl will also lead to higher DSRF expression. However, by imunohistochemical analysis we could not determine if this is the case. Either way, we have shown that Hh-induced changes in DSRF levels of expression are not responsible for the lack of migratory ability of *ptc* mutant cells.

### Tissue-specificity of Hh-dependent *bnl* regulation

We show that the transcription factor Sr is the effector of Hh signalling responsible for increased *bnl* levels in *bnl*-expressing cells. We propose that this effect is permissive rather than instructive, because we did not detect higher levels of *bnl* expression in all cells receiving the Hh signal or in those with higher levels of *sr* expression. This proposal is supported by previous results showing that general ectodermal expression of *sr* does not lead to the general expression of *bnl* [46], [54]. In view of these observations and our own results, we suggest that this regulation is achieved cell-specifically. Essentially, this means that complete regulation of *bnl* expression requires another tissue-specific factor that can synergize with Sr in order to fine-tune its transcription levels (Fig. 8). Our proposed model is supported by the idea that many cell signalling pathways such as Hh, Notch and Receptor Tyrosine Kinase (RTK) are characterised by activator insufficiency, meaning that pathway activation is necessary, but not sufficient, to fully induce their transcriptional targets [15]. Furthermore, we propose that *bnl* expression is induced by Hh signalling by cooperative activation, combining a signal-regulated transcription factor (Sr) with a local activator (X).

What would such complexity in *bnl* transcription regulation accomplish? Transcription is a highly regulated process involving the combined interaction of many factors that control the selective activation or repression of specific genes. These regulated interactions ultimately allow a sophisticated response to multiple environmental conditions, as well as control of spatial and temporal differences in gene expression. Sr could be the factor responsible for this control in cells that already have the machinery required, but not sufficient, to express *bnl*. Sr is the *D. melanogaster* homologue of the Early Growth Response (EGR) vertebrate transcription factors [57]. Amongst the characteristics of mammalian EGR genes are their capacity to be rapidly induced and their ability to form heterodimers to regulate gene expression [58]. Therefore, we can envisage a scenario where Sr, together with another factor (we called X in our proposed model, in Fig. 8), is responsible for the spatio-temporal fine-tuning of the regulation of *bnl* expression. Because the expression of this gene is not Hh-dependent in all cells, we extrapolate from our observations that the mechanism leading to the spatio-temporal regulation of *bnl* transcription involves combinatorial control by Hh-sensitive (like Sr) and Hh-independent transcription factors.

### Early growth response (Egr) genes in the spatio-temporal regulation of gene expression

We show that Hh signalling leads to the induction of *sr* expression and that this transcription factor modulates *bnl* expression. *sr* is required for determining the cell fate of muscle attachment sites that mark the segment border of the embryo in response to Ci activation [44], [45]. *sr* is expressed in muscle-attachment cells, which are responsible for providing guiding cues for myotube migration [43]. These ectodermal cells generate long-range signals that are involved in myotube guidance and that determine their spatial orientation [43], [45]. It would be of interest to determine whether one of these signals is *bnl* and whether this growth factor is also involved in myotube guidance.

Sr is homologous to the Egr family of transcription factors [57]. These transcription factors regulate many genes encoding proteins of diverse functions within various cell types. A characteristic of the mammalian Egr genes is that they are rapid and transiently induced by many mitogenic and differentiation-inducing intercellular signals [59], [60].

Egr-1, first discovered as a gene rapidly induced in response to serum [61], has been shown to regulate FGF2 levels during angiogenesis and tumour growth [62]. This type of rapid regulation is also present in the immune system where Egr-1 is involved in cytokine expression [58]. In addition, the gene expression of the Egr family is also associated with the rapid activation of neuronal plasticity-associated transcription [60]. Overall, the Egr family of transcription factors is involved in responses to physiological stimuli involving rapid changes in gene transcription. It is therefore reasonable to hypothesise that the Sr participates in the rapid response to Hh signalling necessary for the swift changes in *bnl* expression that are required for tracheal cell migration.

## Conclusions

This study reveals a direct connection between Hh signalling and the induction of *bnl* expression, which is accomplished through the transcription factor Sr, together with another unknown cell-specific factor. Cell migration in response to FGF signalling must be carefully regulated and coordinated with other cellular processes. This delicate balance is probably maintained by tight control of the temporal and spatial expression patterns of Hh targets and other cell-specific molecules. Furthermore, both the absence of Ptc-1 or deregulation of FGF/FGFR activities are hallmarks of many human cancers accompanied by higher cell motility and invasiveness [12], [48]. For these reasons, it is likely that the mechanism proposed here for this rapid induction of Bnl/FGF is also conserved in vertebrates.

## Methods

### *D. melanogaster* stocks and genetics

The following stocks are described in FlyBase (http://flybase.bio.indiana.edu): Df(2R)ED1742, *sim*Gal4, *twi*Gal4, srGAL4, UASsr, 69BGAL4, and UASCD8GFP, *hh* ^21^, *sr^155^*. We also used *ptc* ^IIw^, *ptclacZ*, *UAS-Hh* and *UAS-Hh-Ptc* (from A. Casali, Barcelona, Spain), *btlmoeRFP* (from M. Affolter, Basel, Switzerland), *UASCi* (from T. Orenic, Chicago, USA) and *Complex2* (from C. Samakovlis, Stockholm, Sweden). Wild-type is *yw* and *ptc* refers to *ptc^D130^* unless otherwise stated. We used the GAL4 system [63] for over or misexpression experiments. *insc*Gal4 (from G.Tear, London, UK) was used as a VNC driver, *btl*GAL4 (from M. Affolter) as a general tracheal driver, *ptc*GAL4 (from A. Casali) as a *ptc*-positive cell driver. Heterozygous embryos were recognized by lacZ and GFP balancer chromosomes. *D. melanogaster* stocks and crosses were kept under standard conditions at 25°C. All overexpression experiments were conducted at 29°C.

### Immunohistochemistry, image acquisition and data analysis

Embryos were staged as described by Campos-Ortega and Hartenstein [64] and stained following standard protocols. For immunostaining, embryos were fixed in 4% formaldehyde for 20-30 minutes. We used antibodies that recognise GFP (Molecular Probes and Roche), RFP (abcam), ßGal (Cappel and Promega), Ptc (from A. Casali), DSRF (Active Motif), Dof (from M. Leptin, Cologne, Germany), Trh (from J. Casanova, Barcelona, Spain), SrB (from T. Volk, Rehovot, Israel) and 2A12 (DSHB). We used HRP, Alexa488, Alexa-555 and Alexa-647, Cy2, Cy3 and Cy5 conjugated secondary antibodies (Jackson ImmunoResearch). For HRP histochemistry, the signal was amplified using the Vectastain-ABC kit (Vector Laboratories) when required. In addition, the signal for the DAB reaction was intensified with NiCl_2_, except for double stainings, where it was omitted from one of the reactions. For tracheal lumen visualisation, we also used fluorescently-conjugated CBP (NEB). *In situ* hybridisation was performed following standard protocols. ribo-*bnl* was generated using the whole cDNA as a template and using the Megascript kit (Ambion). Photographs were taken using a Nikon Eclipse 80i microscope. Fluorescent images were obtained with confocal microscopes (Leica TCS-SPE-AOBS and Leica TCS-SP5-AOBS system) and processed using Fiji [65] and Adobe Photoshop (Adobe Systems) softwares. Images are maximum projections of confocal Z-sections. Phenotypes were scored using Nomarski optics on a Nikon Eclipse 80i microscope with a 20x or 40x objective. Cells were counted using Imaris x64 7.6.1 (Bitplane) software.

### Time-lapse experiments

*btl*Gal4UAS-*CD8*GFP (Movies 1 and 2) embryos, wt or *ptc* mutant, were mounted for live imaging as described [66]. Z-stacks (0.5 μm step size) were collected on a Leica TCS-SP5-AOBS system and processed using Fiji [65]. Sections were recorded every 3 minutes for 6–8 hours.

### Real-Time PCR

Real-time PCR was performed with a Roche LightCycler 480 using the SYBR Green I method following the manufacturer's instructions. The two sets of primers for *bnl* and for the *rp49 control* were designed on exon-exon junctions to avoid genomic amplification, using the Primer Express software (Applied Biosystems). To assess levels of expression, we extracted total RNA from embryos with Trizol reagent (Invitrogen) following the manufacturer's instructions. *ptc* homozygous mutant embryos were separated by means of a GFP balancer. We prepared cDNAs with the RevertAid H Minus First Strand cDNA Synthesis Kit (Fermentas) using random hexamer primers. The nature of the PCR products was confirmed by melting curve analysis. All analyses were performed using the Relative Expression Software Tool (REST) following the user manual and references. The main steps of the automatic REST workflow were as follows: PCR efficiencies were calculated for each pair of primers by generating standard curves at increasing dilutions of cDNA (1∶2, 1∶4, 1∶8, 1∶16) and used to correct raw data. *rp49* was assumed to be equally expressed in wt and mutant/overexpressing embryos and was used as a reference to normalise data. A ratio between the normalised signals of tested genes in mutant and wt embryos was calculated and expressed as fold increase/decrease, and statistically tested by a bootstrap test (10000 randomisations). We used a sample size of 3 repeats per plate, for each embryonic genotype tested, using a set of primers for *bnl*, and each experiment was repeated twice.

## Supporting Information

Figure S1 **Different allelic combinations of** ***ptc*** **have the same phenotype and inhibition of apoptosis does not change the GB phenotype.** (A–E) Stage 16 wt (A), *ptc^IIw^* mutant (B), *ptc^D130^* over the deficiency that deletes *ptc* (C), *ptc^D130^* over the deficiency that deletes *grim*, *reaper* and *hid* (D) and *ptc^D130^* heterozygous embryos stained with the tracheal lumen antibody 2A12, using HRP immunohistochemistry for visualization. A B, C, D and E are dorsal views; A′, B′, C′, D′ and E′ are lateral views and A″, B″, C″, D″ and E″ are ventral views. (F) Sequence comparison between the wild-type and *ptc* D130 mutant; detection of the base difference that leads to an early STOP codon. Represented are nucleotides 1918 to 2067 of the *ptc* cDNA.
(TIF)

Figure S2 **Ptc is expressed in cells surrounding the migrating tracheal branches and inactivation of the Hh pathway does not affect GB migration.** (A–F) Different stage embryos expressing *ptclacZ* stained with anti-ßgal and anti-Trh. Ptc expression is detected by nuclear βgal presence. Scale bars are 10 μm. Panels B, D and F show a single Z-section. (G–I) Ventral views of stage 16 embryos stained with 2A12 to visualize the tracheal lumen.
(TIF)

Movie S1 **Wild-type GB migration and generation of filopodial extensions.** wild-type embryo carrying *btl*GAL4 and UAS*CD8*GFP constructs to reveal tracheal cell morphology and cytoplasmic extensions. One tip-cell is present at the tip of each GB as it migrates towards the VNC. This cell extends long filopodial extensions in response to FGF signalling.
(AVI)

Movie S2 ***ptc*** **mutant GB migration and generation of filopodial extensions.** *ptc* mutant embryo carrying *btl*GAL4 and UAS*CD8*GFP constructs to reveal tracheal cell morphology and cytoplasmic extensions. One tip-cell is present at the tip of each GB as it tries to migrate towards the VNC. This cell extends long filopodial extensions in response to FGF signalling, but is not able to advance in the VNC direction.
(AVI)

Tables S1 **Supporting tables.** Table S1, qRT-PCR Results. Table S2, qRT-PCR Results.
(DOCX)
