# Supplementary figures and images for: Hedgehog Is a Positive Regulator of FGF Signalling during Embryonic Tracheal Cell Migration
Source: PLoS One. 2014 Mar 20;9(3):e92682. doi: 10.1371/journal.pone.0092682 (PMC3961400; doi:10.1371/journal.pone.0092682)

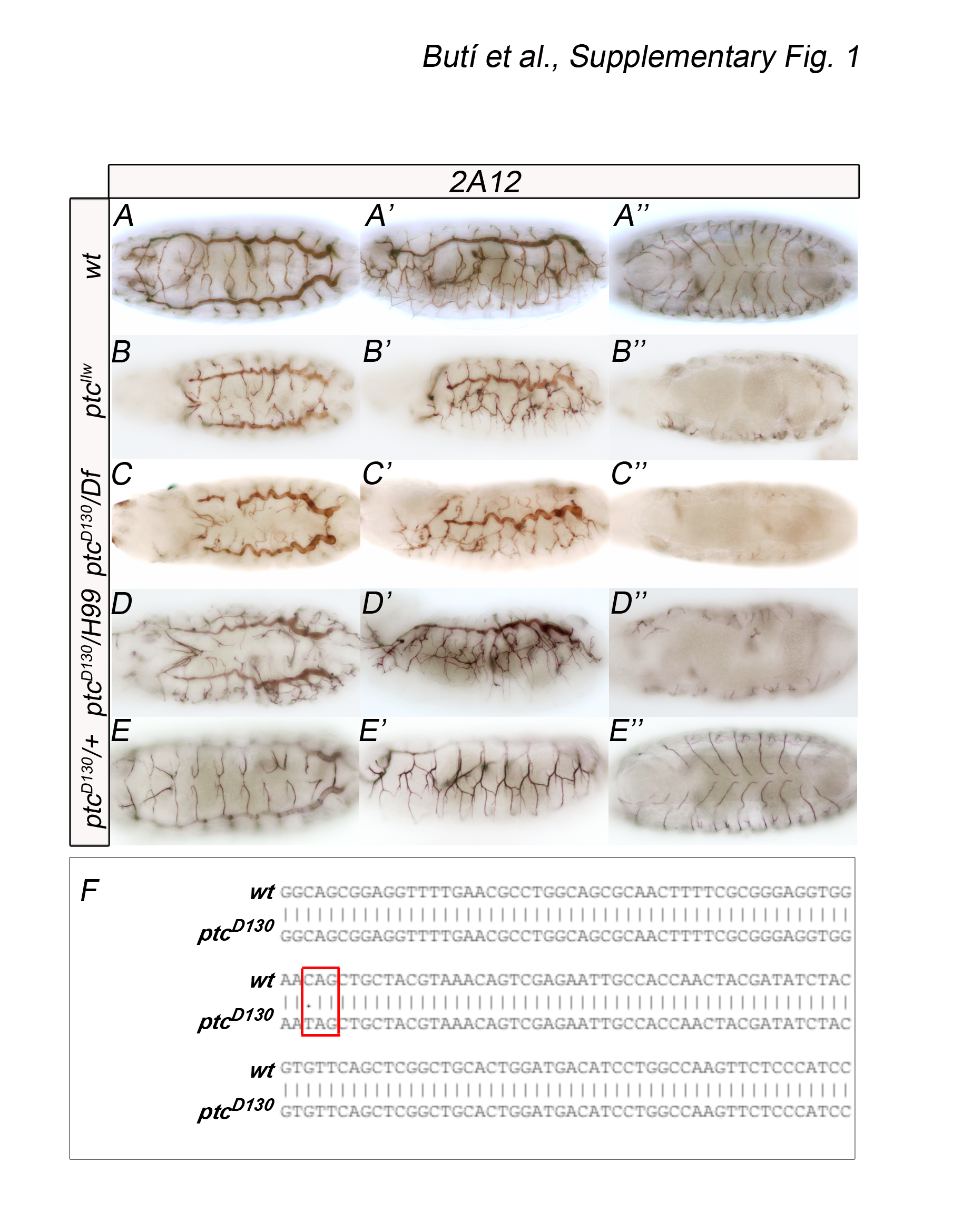

Supplement: Figure S1 — Different allelic combinations of ptc have the same phenotype and inhibition of apoptosis does not change the GB phenotype. (A–E) Stage 16 wt (A), ptcIIw mutant (B), ptcD130 over the deficiency that deletes ptc (C), ptcD130 over the deficiency that deletes grim, reaper and hid (D) and ptcD130 heterozygous embryos stained with the tracheal lumen antibody 2A12, using HRP immunohistochemistry for visualization. A B, C, D and E are dorsal views; A′, B′, C′, D′ and E′ are lateral views and A″, B″, C″, D″ and E″ are ventral views. (F) Sequence comparison between the wild-type and ptc D130 mutant; detection of the base difference that leads to an early STOP codon. Represented are nucleotides 1918 to 2067 of the ptc cDNA. (TIF) [file pone.0092682.s001.tif]

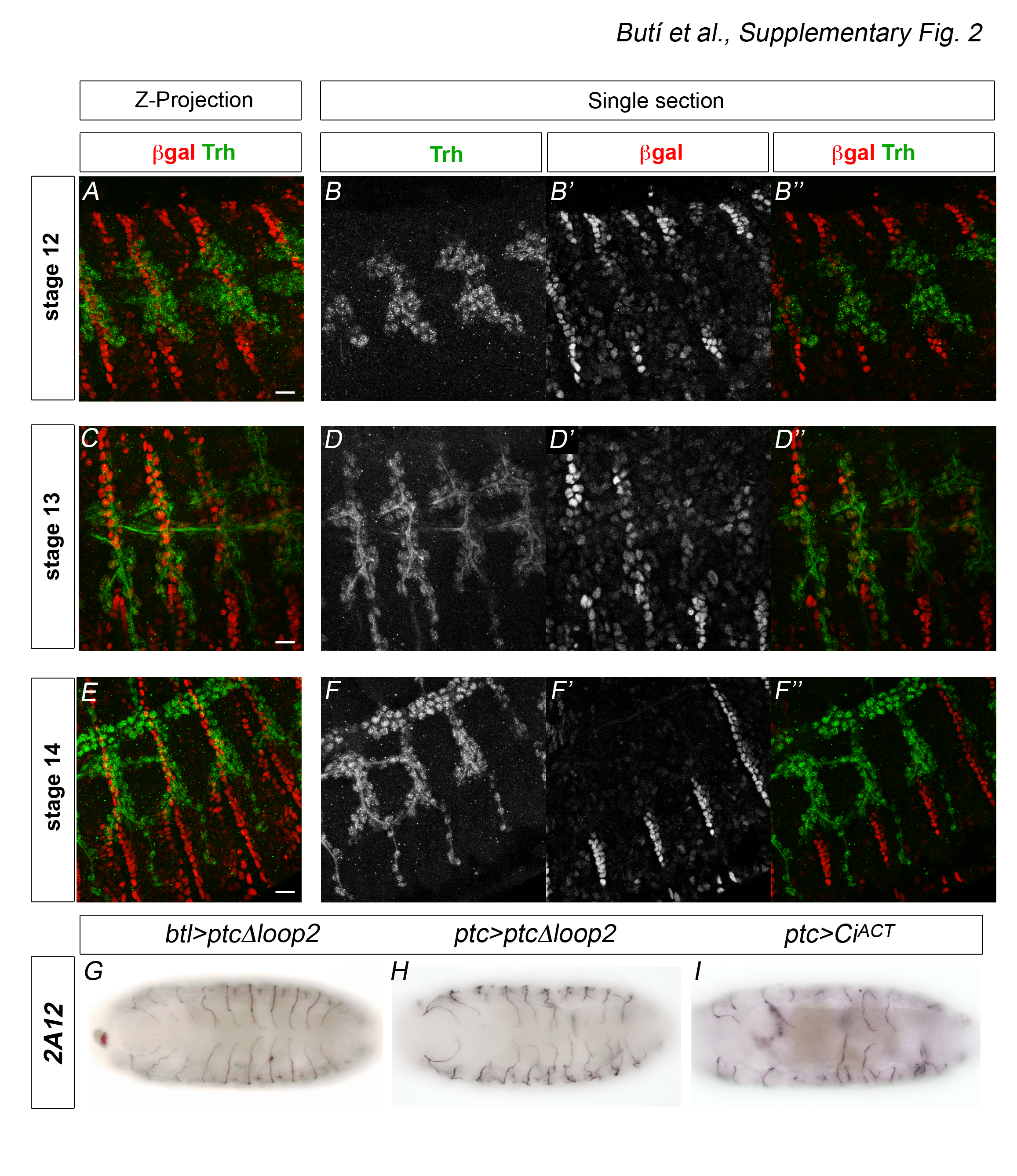

Supplement: Figure S2 — Ptc is expressed in cells surrounding the migrating tracheal branches and inactivation of the Hh pathway does not affect GB migration. (A–F) Different stage embryos expressing ptclacZ stained with anti-ßgal and anti-Trh. Ptc expression is detected by nuclear βgal presence. Scale bars are 10 μm. Panels B, D and F show a single Z-section. (G–I) Ventral views of stage 16 embryos stained with 2A12 to visualize the tracheal lumen. (TIF) [file pone.0092682.s002.tif]
