# Supplementary material for: Hedgehog Is a Positive Regulator of FGF Signalling during Embryonic Tracheal Cell Migration
Source: PLoS One. 2014 Mar 20;9(3):e92682. doi: 10.1371/journal.pone.0092682 (PMC3961400; doi:10.1371/journal.pone.0092682)
Supplement: Tables S1 — Supporting tables. Table S1, qRT-PCR Results. Table S2, qRT-PCR Results. (DOCX) [file pone.0092682.s005.docx]

**E. Butí, D. Mesquita and S. J. Araújo, Supporting information**

**Table S1 – qRT-PCR Results**

|  | wt | *ptc*^D130^ | *insc* > *bnl* | *ptc* > *hh* | *twi* > *hh* |
| --- | --- | --- | --- | --- | --- |
| *bnl* fold increase | 1 | 6.027*  5.278 | 16.745*  12.442 | 2.571*  3.116 | 2.123*  2.040 |
| Interquartile range | - | (3.536-9.332)  (3.892-7.527) | (15.398-18.073)  (9.933-15.109) | (2.413-2.730)  (2.474-3.706) | (1.893-2.408)  (1.581-2.415) |

* values from two independent experiments of 3 repeats per plate each.

**Table S2 – qRT-PCR Results**

|  | wt | *ptc*^D130^ | *69B* > *sr* | *69B* > *CiAct* | *ptc* > *CiAct* |
| --- | --- | --- | --- | --- | --- |
| *bnl* fold increase | 1 | 2.059*  1.782 | 2.185*  2.010 | 3.462*  2.925 | 2.219*  2.175 |
| Interquartile range | - | (1.929-2.255)  (1.359-2.268) | (1.740-2.512)  (1.636-2.436) | (3.144-3.826)  (2.401-3.313) | (2.054-2.394)  (1.737-2.507) |

* values from two independent experiments of 4 repeats per plate each.
